# Most yeast SH3 domains bind peptide targets with high intrinsic specificity

**DOI:** 10.1371/journal.pone.0193128

**Published:** 2018-02-22

**Authors:** Tom Brown, Nick Brown, Elliott J. Stollar

**Affiliations:** 1 Math and Computer Science Department, Eastern New Mexico University, Portales, NM, United States of America; 2 Portales High School, Portales, NM, United States of America; 3 Physical Sciences Department, Eastern New Mexico University, Portales, NM, United States of America; Universita degli Studi di Roma Tor Vergata, ITALY

## Abstract

A need exists to develop bioinformatics for predicting differences in protein function, especially for members of a domain family who share a common fold, yet are found in a diverse array of proteins. Many domain families have been conserved over large evolutionary spans and representative genomic data during these periods are now available. This allows a simple method for grouping domain sequences to reveal common and unique/specific binding residues. As such, we hypothesize that sequence alignment analysis of the yeast SH3 domain family across ancestral species in the fungal kingdom can determine whether each member encodes specific information to bind unique peptide targets. With this approach, we identify important specific residues for a given domain as those that show little conservation within an alignment of yeast domain family members (paralogs) but are conserved in an alignment of its direct relatives (orthologs). We find most of the yeast SH3 domain family members have maintained unique amino acid conservation patterns that suggest they bind peptide targets with high intrinsic specificity through varying degrees of non-canonical recognition. For a minority of domains, we predict a less diverse binding surface, likely requiring additional factors to bind targets specifically. We observe that our predictions are consistent with high throughput binding data, which suggests our approach can probe intrinsic binding specificity in any other interaction domain family that is maintained during evolution.

## Introduction

**Fig 1:**
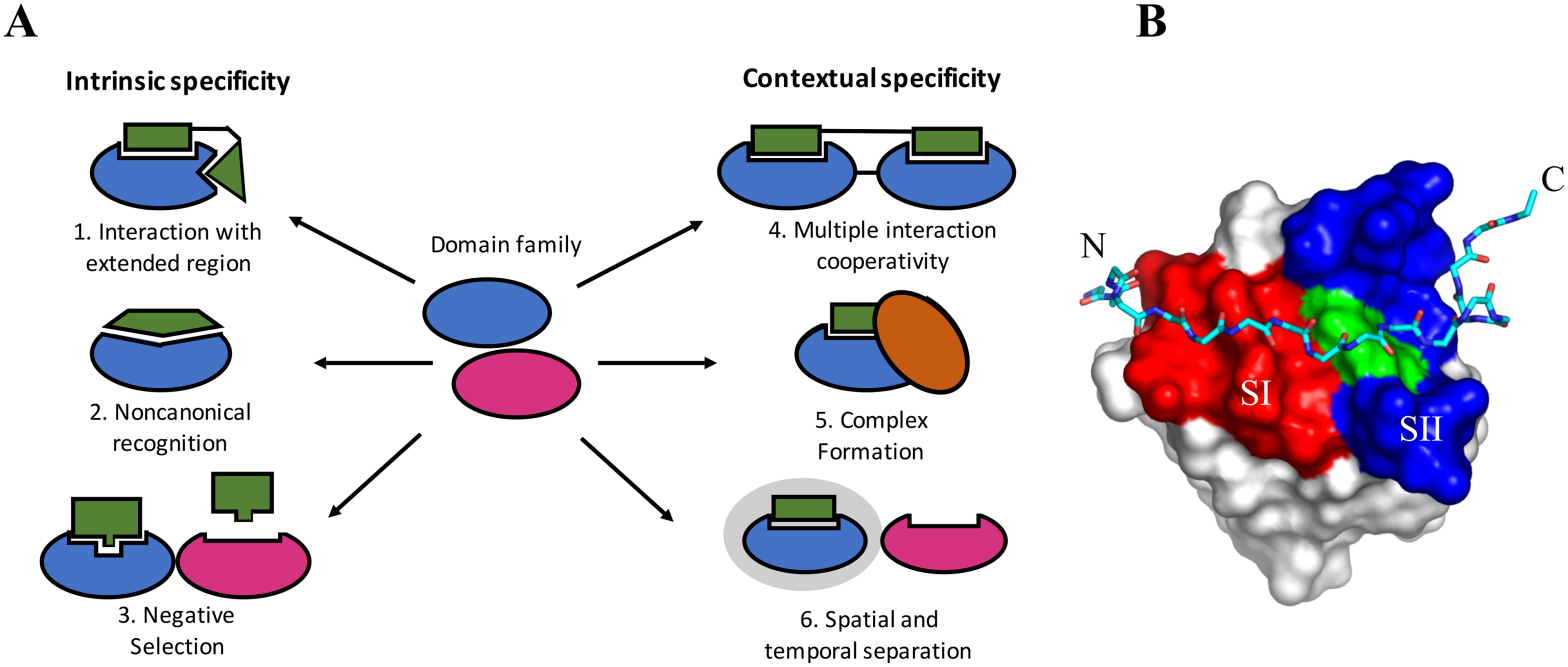
General mechanisms to obtain binding specificity in domain families. A. Domains may use the interaction with an extended region that goes beyond the canonical binding site to obtain intrinsic specificity (1). For example, the Abp1p SH3 domain binds extended target peptides (17 residues) and was shown to possess high intrinsic binding specificity [9, 10]. Domains may also achieve intrinsic specificity through non-canonical recognition via an alternative binding surface far from the canonical one. For example, Pex13 is a peroxisomal membrane protein that contains an SH3 domain that binds Pex14p via the canonical binding surface, however, it also binds Pex5p through an alternative non-canonical surface [11, 12]. Furthermore, intrinsic specificity may be achieved through replacing the canonical binding site with a non-canonical one (2) that would lead to negative selection (3) with respect to proline-rich peptides that bind SH3 domains. For example, Fus1 peptide targets do not contain a canonical PxxP motif thus minimizing cross reactivity to proline containing peptides [13]. Some domains may have potential for contextual specificity using adjacent domains (4). For example, at least 2 of the 3 adjacent SH3 domains of Nck are required to bind their targets [14]. Spatial and temporal separation mechanisms may be another contextual specificity mechanism (6). For example, *in vitro*, Fyn SH3 domain and CD2BP2 both bind and compete with each other for the proline region in the target protein CD2. However CD2BP2 localizes to the cytosolic compartment where it interacts with CD2 in T-cells, while Fyn is present permanently in the lipid raft fraction unable to compete [15]. In some cases, both intrinsic and contextual specificity mechanisms may be used by a domain, such as the Pex13p example above (5). We note here that contextual specificity has been used elsewhere to mean the extended regions of SH3 domain binding peptides, outside their core binding motif [16]. This definition does not pertain to contextual specificity as discussed within this study. Figure adapted from [17] and [7]. B. An example of an extended peptide-domain interaction. The Ark1 peptide is represented in stick and the SH3 domain from Abp1 uses space-filling. The red region is surface I and the blue region is surface II. W36 is represented as green and is on the boundary of the two surfaces. Adapted from [18] (pdb code 2rpn).

Signals are transmitted through cellular pathways via relays of protein-protein interactions resulting in specific outputs, such as cell growth, differentiation, or apoptosis. To achieve the correct responses from signaling pathways, the protein-protein interactions involved must be specific, and not potentiate inappropriate activation of off-target pathways. This requisite precision can be readily achieved by proteins that possess high “intrinsic specificity”, directly binding their intended targets much more tightly than any other protein. For protein-DNA interactions, this can involve differences of three orders of magnitude or more in K_d_ value between target and non-target binding [1]. For example, the cro repressor binds its cognate OR3 operator with a K_d_ of 2 pM while binding non-specific DNA ∼ 10^4^ times weaker with a K_d_ of 1.5 *μ*M [2]. However, other proteins appear to have low intrinsic specificity, binding their intended target and many other non-specific targets with similar affinities [3–5]. For example, Michaud *et al*. analyzed the binding of 11 antibodies to ∼5000 different yeast proteins and although they found five were highly specific towards their antigen, five others were cross reactive towards a number of other antigens, and one was promiscuous, binding >1000 partners [6]. The interactions of these proteins may still achieve high specificity through alternative mechanisms that Bhattacharyya *et al*. define as “contextual specificity” [7]. Contextual specificity is the contribution of the environment to interaction specificity. For example, the intended target can be separated from other proteins through coordinated temporal and spatial localization within the cell. This is seen in the case of signaling pathways that are initiated at the membrane, where recruitment serves to enhance specificity by increasing the local concentration of the specific interaction partners over other proteins. Contextual specificity also operates through the requirement that some target proteins bind in a cooperative multi-protein complex. As such these proteins usually provide additional binding sites in the interaction that are less likely to be present in other proteins. Fig 1A illustrates these specificity concepts and provides examples from known SH3 domain interactions. The relative importance of intrinsic and contextual specificity in families of related proteins has not yet been well defined [7, 8], and is the purpose of the present study.

The bulk of protein interactions within signaling pathways are mediated by small modular domains, which are found within larger proteins [19]. SH3 domains are one of the most frequently occurring protein-protein interaction modules in eukaryotic cells and are an excellent model system to address mechanisms of protein binding specificity (Fig 1B). Most of these domains are composed of ∼60 residues, and are primarily *β*-sheet in their secondary structure. SH3 domains generally bind short proline-rich peptides containing the core consensus sequences +xxPxxP (class I) or PxxPx+ motifs (class II), where x can be a variety of residues while + is a Lys or Arg residue [20–22]. The canonical SH3 domain surface I (SI) interacts with a peptide PxxP motif and is comprised of two shallow hydrophobic grooves. These grooves are formed primarily by the conserved residues, Tyr/Phe8, Tyr/Phe10, Trp36, Pro51 Asn53 and Tyr/Phe54 (red surface in Fig 1B) which binds a myriad of target peptides with modest affinities (5-100 *μ*M) [23].

Early studies with short PxxP-containing peptides that bind predominantly to SI, showed SH3 domains have similar target peptide affinities [24–28], suggesting that they may depend on contextual specificity. For example, the Grb2-Sos interaction requires cooperative binding of both the C and N-terminal Grb2 SH3 domains with one of four Sos PxxP sites to enable a specific interaction [29, 30]. Similar to the Grb2-Sos interaction, many SH3 domain containing proteins contain more than one SH3 domain, providing multiple potential binding surfaces for peptides to attain contextual specificity (in yeast, Bem1p, Sla1p and Bzz1p contain multiple domains). There also exist a few examples of SH3 domains recognizing targets through a completely different surface to SI, such as Pex13p [11, 12]. This raises the possibility that these domains can mediate multiple interactions through different binding surfaces on the same domain [31–33], providing yet another mechanism for attaining contextual specificity. Finally, in addition to binding peptides, SH3 domains have been found to bind folded domains. For example, SH3 domains of several endocytic proteins, including the yeast Sla1p, mammalian CIN85 and amphiphysin proteins, have been shown to interact with ubiquitin [34]. Interestingly, these interactions also engage the conserved SH3 domain residues of SI involved in canonical peptide interactions further suggesting the need for contextual specificity mechanisms in these cases.

Despite these examples, it has been shown that the peptide residues flanking the PxxP motif can play crucial roles in mediating binding, and provide support for intrinsic specificity in SH3 domain interactions [35–37]. These flanking peptide residues lie N-terminal to the PxxP motif in class I peptides and C-terminal in class II peptides, are variable in sequence, and interact beyond SI with a broad SII located between the RT- and N-Src loops [38] as shown by the blue surface in Fig 1B. The recognition of extended peptide sequences by these domains implies their level of intrinsic specificity will be higher than those recognizing shorter sequences. This notion is supported by the ability of the extended peptide to also make interactions with non-canonical surfaces that are adjacent to the canonical binding surface, as seen in Abp1p for example (Fig 1B). This is especially true when the extended interface is unique within the domain family for a given species. In the case of Sho1p and Abp1p SH3 domain, biologically relevant extended target peptides were shown to possess high intrinsic binding specificity and decreasing the level of binding specificity was detrimental to the fitness of the cell [9, 10]. Recent studies with high-throughput binding assays using phage display consensus sequences, show the majority of yeast SH3 domains have unique preferences for their target peptides. Many of these go beyond the basic core PxxP motifs and appear to be conserved over evolution [39–41]. These results suggest extended peptide regions may play an important role in many SH3 domain-peptide interactions and provide a mechanism for intrinsic specificity. Furthermore, this may be predictable through sequence and structural analysis, if it can be shown that a given domain in a family has a unique surface in this extended region.

As such, we hypothesize that the analysis of domain sequence alignments can predict whether each yeast SH3 domain binds its targets with intrinsic and/or contextual specificity, which will be compared to published binding and structural data. Our approach is motivated by the numerous fungal genomes that have been sequenced [42] and the observation that there appears to be a general correlation between domain sequence identity and binding specificity, based on top binding peptide consensus sequences [41]. Furthermore, complimentary to yeast SH3 domain phage display studies [40, 41], through a computational approach Kelil *et al*. show peptide target conservation is correlated with peptide binding specificity for the complete yeast SH3 domain family [43]. Thus, to complement target peptide sequence analysis, we study domain conservation and structure to show if predictions can be made about domain function in the absence of peptide binding data.

We compare the sequences of every yeast SH3 domain in a paralog alignment, which reveals the key conserved features for any SH3 domain (basic/common fold and function). We also compare the conservation of each domain family member with its direct relatives in an ortholog alignment, to highlight the key conserved and unique features for each family member. Those positions that are conserved in both paralog and ortholog alignments indicate the housekeeping residues for essential SH3 domain structure and function. However, positions conserved in the ortholog alignment, but not conserved in the paralog alignment, have high specific conservation (SC) and suggest they provide uniqueness to that domain. We find that most SH3 domains in yeast have high SC in SII, suggesting they have the capacity to bind with intrinsic specificity. Additionally, some of the studied SH3 domains have high SC in alternative regions suggesting other important binding surfaces may exist. Comprehensive sequence alignment analysis of the yeast SH3 domain family promises to provide greater understanding of how its members mediate interactions inside the cell.

## Materials and methods

### Overall sequence alignment analysis

The National Center for Biotechnology Information (NCBI) protein database was queried to generate paralog information (find all SH3 domains in *S.cerevisiae*) and ortholog information (find direct relatives for a given domain). All alignments are outputted as formatted excel spreadsheets. In all alignments, the following 6 amino acid equivalency groups are used to calculate the entropy [44] at each residue position using Eq (1); AVLIMC (1), FWYH (2), STNQ (3), KR (4), DE (5), GP (6).

$$\begin{array}{c} Positional\;entropy=e^{-\sum_{i=1}^{6}p_{i}ln\left(p_{i}\right)}, \end{array}$$ (1)

where *p*_*i*_ is the fraction of residues that belong to that equivalency group (i = 1, 2, 3, 4, 5 or 6). For positions in the alignment where there are too many gaps (defined by “% no gap threshold”), entropies are not calculated. The threshold is 0.64, which corresponds to residues present in at least ∼19 of 29 sequences.

### Paralog alignment analysis

The NCBI protein database was searched using the ENTREZ Global Query Cross-Database Search System for all entries that contains both the domain family name (SH3) and organism name (saccharomyces cerevisiae S228c). From this information, we extract/identify the domain family members (paralogs) from the ENTREZ records and use ClustalW [45] to perform a multiple sequence alignment of the domains. Positional entropies are calculated and standard domain numbering [46] is added to the paralog alignment manually as well as making small manual alignment adjustments before running the ortholog alignment analysis (S1 File). It should be noted that some of the proteins have the following common synonyms, Boi1 or Bob1, Lsb4 or Ysc84, Scdc25 or YL017, Cyk2 or Hof1 and Lsb2 or Pin3)

### Ortholog alignment analysis

We examine the evolution of each of the 28 yeast domain family members within the fungal kingdom by generating an ortholog alignment for each. We first perform a separate protein BLAST on the NCBI database for each of the 28 full length protein in *S.cerevisiae* using an e-value filter (measure of protein similarity) of less than 1e-5. In almost all cases, this filter selected true orthologs to the query protein. Next, we performed an ENTREZ text search for each protein to extract the protein length and the location of their SH3 domains. For yeast proteins that have multiple SH3 domains, a ClustalW alignment is performed between the known yeast domain and the multiple domains found in the given ortholog and the closest matching domain is retained. In some cases, manual adjustments were made after this procedure to ensure the relative order of the domains was maintained. We found ∼250 ortholog species for each yeast domain, and reduced redundancy by the following method. For every species containing SH3 domains, we counted the number of different domain family members found in that species and referred to this as the species paralog count (SPC). Some species had as few as 1 yeast SH3 domain direct relative, while many had SPC values close to 28, which is the number of domain family members in *S.cerevisiae*.

To construct the final ortholog alignment, proteins were chosen that satisfy the distribution in our phylogenetic tree (S1 Table and S1 Fig), while using species with as high an SPC value as possible yet having diverse lineages from each other within that taxonomy group. As such, ancestral SH3 domains were chosen, that maximizes diversity and minimizes redundancy (S1 Fig). This is a critical component of our approach and takes advantage of the tremendous 1 billion year evolutionary span and numerous sequenced genomes in the fungal kingdom [47]. For 5 domain family members, there are less than 29 sequences in their ortholog alignments as they have insufficient representation in 1 or more of the taxonomic groups (S2 Table). For each domain, the orthologs selected in the previous step are aligned, visualized and analyzed in two alignment files. One alignment is adjusted to show only the positions that align with the given *S.cerevisaie* SH3 domain (S1 File), while another shows the complete protein sequences (S2 File). Ortholog positional entropy is calculated at each residue position according to Eq (1). For the domain alignment, the paralog entropy values at the corresponding positions is retrieved from the paralog alignment and the paralog/ortholog entropy ratio for each position is also calculated. We refer to this value as a specific conservation (SC) value, where a high number reflects higher conservation in the ortholog alignment than the paralog alignment. The amino acids are colored in S2 and S1 Files according to the residue equivalency groups defined for the entropy calculations in Eq (1).

### Peptide binding data analysis

Binding data (normalized SPOT binding intensities) between ∼300 peptides and the SH3 domain family members from 4 fungal species was recently collected and has been analyzed further [37]. Before calculations, the following domain paralogs in a given species have their binding intensities averaged and value used only once, *C.albicans* Abp1 (2 domains), *A.gossypii* Bem1a (2 domains), *C.albicans* Bem1a (2 domains), *S.pombe* Hof1 (3 domains), *S.cerevisiae* Lsb1/Lsb2 (2 domains), *S.cerevisiae* Lsb3/Lsb4 (2 domains), *S.cerevisiae* Myo3/Myo5 (2 domains), *S.cerevisiae* Boi1/Boi2 (2 domains) and C.albicans Rvs167 (2 domains). For a given peptide i, binding to a given domain d1, from a given species j, we calculated a binding fraction (BF) using binding intensity (BI) data for peptide i to all domains in that species. As listed above, 4 domain pairs in *S.cerevisiae* are averaged and thus BI’s from 24 domains are considered (d1 to d24)). Eq (2) calculates the BF for domain 1 (d1) and a similar equation is used to calculate BF’s for the other domains in the family.

$$\begin{array}{c} BF\left(i,j,d1\right)=\frac{BI(i,j,d1)}{\sum_{k=d1}^{d24}BI\left(i,j,k\right)}, \end{array}$$ (2)

We do not calculate BF’s for any peptide that has a sum of intensities to all domains in a given species below 1000 units to ensure good signal over noise and minimize inflated proportions from small numbers in the calculation for Eq (2). We define a binding fraction of greater than 0.5 to be a specific domain-peptide interaction within a given species. The specific peptide sequences identified for the yeast SH3 domain family can be found in S3 File.

### Position Specific Scoring Matrices (PSSMs) and Clustering

PSSMs contain the number of occurrences for an equivalency group (Eq (1)) at a particular alignment position and were generated for all 28 yeast SH3 domain ortholog alignments. The number of occurrences in these matrices were converted to frequencies that sum to one for each alignment position. A master matrix (28 x 360) was constructed by representing all the relative frequencies for the 6 equivalence groups for each residue (1-60) in a single column for each domain. KMeans clustering algorithm version 0.19.0. from scikit-learn [48] grouped domains in the family using the master matrix according to residues that define SI (8, 9, 10, 36, 37, 51-54) or SII (13-17, 30-35, 38, 49).

## Results

### Specific conservation analysis reveals a unique SII for the majority of yeast SH3 domains

**Fig 2:**
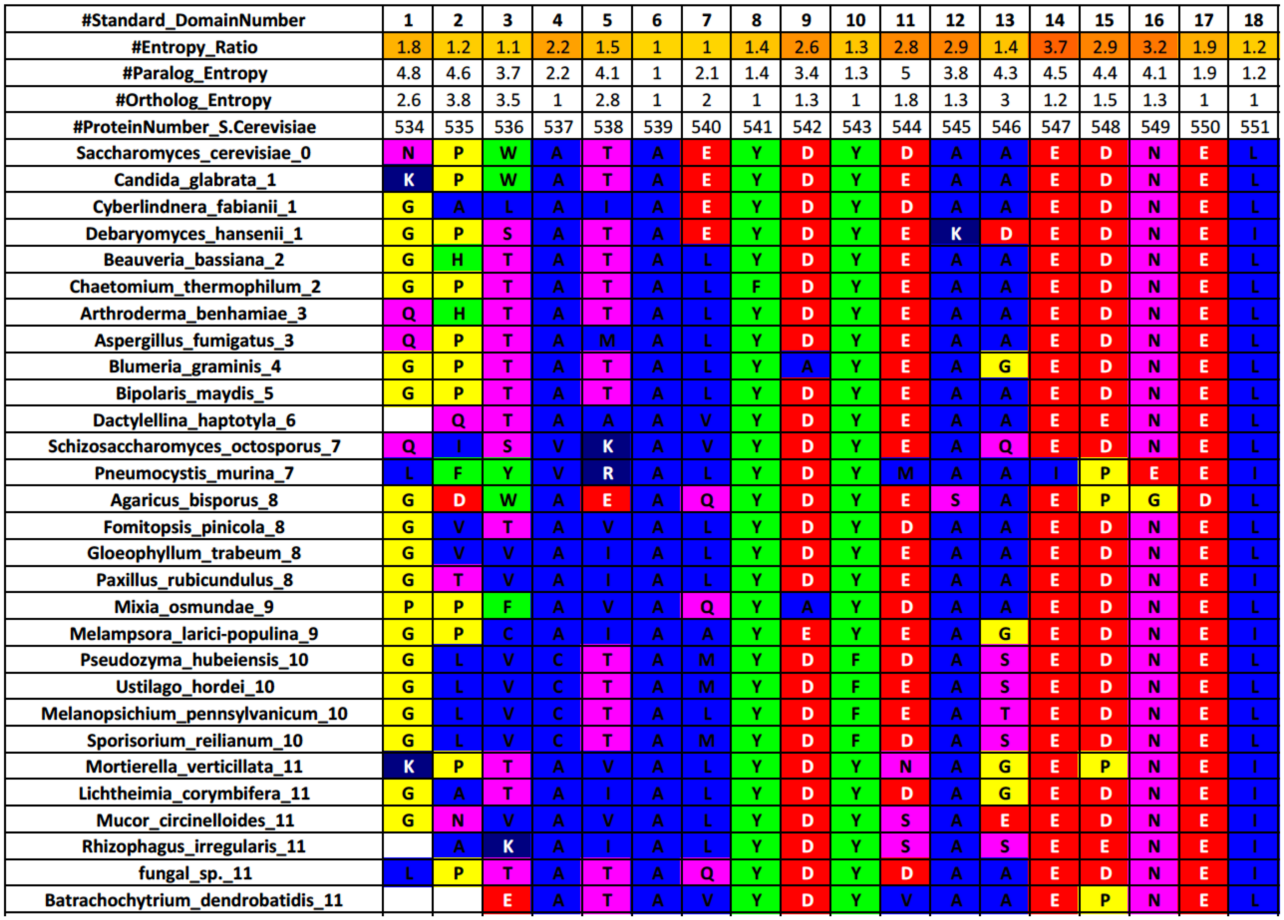
Example sequence conservation analysis for orthologs of Abp1 SH3 domain. The residues are colored according to the residue equivalence groups defined for entropy and PSSM calculations. The species names end with a number that refers to their taxonomic group (S1 Fig and S1 Table). The SC value is calculated as (paralog entropy)/(ortholog entropy). A standard numbering system [46] for the core 60 SH3 domain residues is indicated on the top row as well as the residue number in the full length *S.cerevisiae* protein (fifth row). The paralog entropy is calculated from an alignment of the 28 SH3 domains in *S.cerevisiae*.

To test our hypothesis, we constructed alignments for each yeast SH3 domain by systematically selecting representative species from the >800 fungal genomes sequenced [47]. For each yeast SH3 domain family member (Fig 2 and S1 File), we calculated the positional entropy values for their alignments. We define a positional entropy of n at a given position is equivalent to n groups occurring there with a frequency of 1/n. As a specific example, if a position has a positional entropy value of 4, this is equivalent to four different groups occurring at the position, all with equivalent frequencies of 25% or $\frac{1}{4}$, thereby higher conservation will generate lower entropy values [46]. The 60 canonical SH3 domain residues are defined per numbering from a previous study [46] and for these positions the Specific Conservation (SC) value is calculated (paralog/ortholog positional entropy ratio). The SC value is a simple concept that allows one to assess the uniqueness of any residue position in the domain compared to the overall family. The SC value indicates for a position, how many times more “conserved” it is amongst the orthologs compared to the paralogs. For example, a high SC value for a position in a given domain indicates relatively little conservation amongst paralogs and high conservation amongst orthologs that may distinguish this residue as important for the given domain. When groups of these high SC value residues cluster on the structure (at a binding surface for example), this further supports their contribution to the uniqueness for this domain. As an example, Fig 2 shows the first 18 residues of the Abp1 SH3 domain (a similar file is made for every domain). In this example, position 14 has the highest SC value (3.7), with an ortholog entropy of 1.2 (highly conserved) and a paralog entropy of 4.5 (little conservation). Complete analysis of ortholog alignments can be found in S2 and S1 Files.

**Fig 3:**
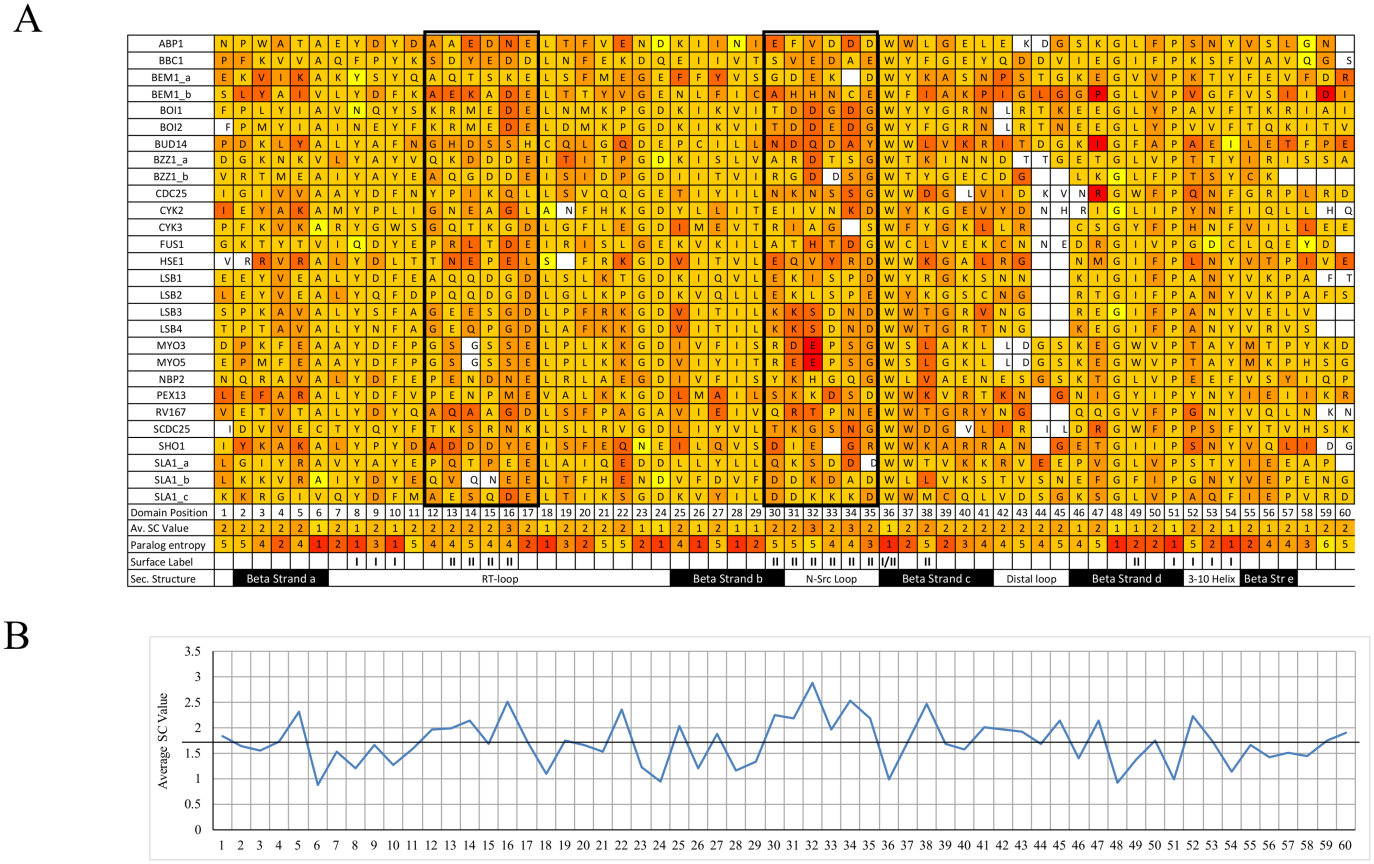
Specific conservation values for the yeast SH3 domain family. A. Alignment of the core 60 positions colored by ortholog SC values as a heat map (red high and yellow low SC values, with domains sorted alphabetically). The average SC value across the family is indicated for each position at the bottom of the table, along with the paralog positional entropy, surface labels and secondary structure. Dark Boxes indicate the 2 principal loop regions where high SC values are found. B. Specific conservation across the domain. The line is set at an SC value of 1.7, which is considered a potential threshold for significant specific conservation (where ortholog conservation is almost twice that of paralog conservation).

We consider the SC values from every domain in the yeast family (Fig 3) and find the positions with the highest SC values are position 32 in the center of the N-Src loop, followed by position 16 in the center of the RT-loop with average SC values of 2.9 and 2.5 respectively (see boxes in Fig 3). From an evolutionary perspective, changes in the middle of flexible SII binding loop regions would be most suitable for acquiring important domain-specific sequences as these regions are less likely to affect the fold or stability of the protein [38]. Several members of the yeast SH3 domain family have insertions in these loops as well as the distal loop and a few members have deletions as well, likely creating even more diversity for the family. For example, Sho1 and Sla1b both have 2 conserved insertions in the RT-loop, Boi1 and Pex13 have 3 conserved insertions in the N-Src loop and Bzz1b has a conserved 4 residue deletion at the C-terminus of the domain.

**Fig 4:**
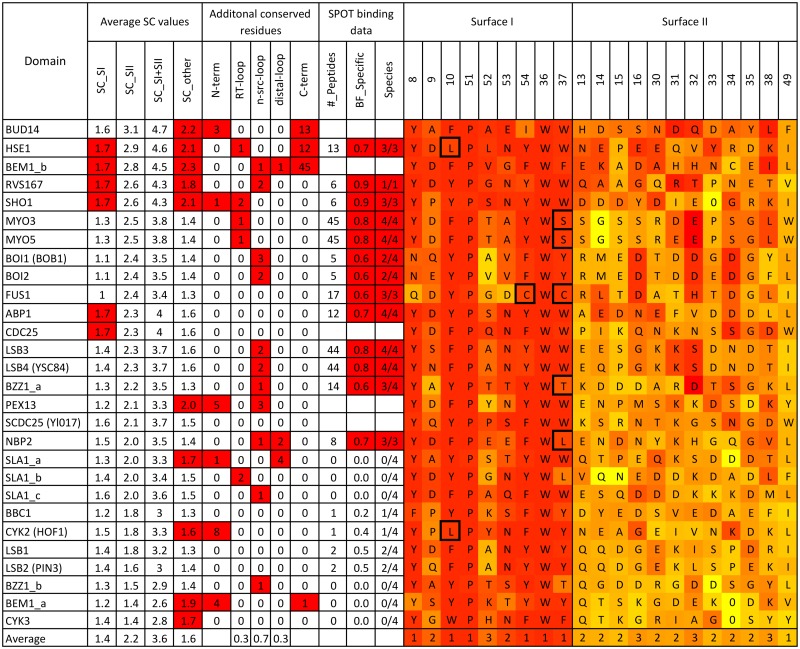
Summary of sequence conservation found in the yeast SH3 domain family. Both SI and SII alignments on the right are heat map colored by either ortholog entropy (SI) or SC values (SII), where red represent high or significant values and yellow as non-significant. As such, we define high average SC values for SI, SII and other (all other residues except SI and II) when ≥ 1.7. We define and count significant additional insertions at the N- and C-terminus, RT-loop, N-Src loop and distal loop when ortholog positional entropy values are ≤ 3.3. Information about the specific binding peptides identified from published binding data [41] is also indicated in the following 3 columns. The “#_peptides” column is the total number of specific peptides where at least 1 species domain family has a binding proportion ≥ 0.5. The “BF_specific” column is the average binding fraction for the most specific (best) peptide across available species. The “Species” column contains 2 numbers, the first is the number of species where the most specific peptide has a binding fraction ≥ 0.5. The second is the number of species where binding data could be collected. Gaps in binding data, indicate the domain was difficult to purify for 2 or more species. Interestingly, from this dataset, known biological peptides targets are sometimes ranked higher for a given domain according to binding fraction values as opposed to binding intensity values (the method used by the authors of the high-throughput study). For example, the Ark1 peptide target (DKKTKPTPPPKPSHL) for Abp1 [10] ranks 2nd using binding fraction and 10th using intensity alone. In the case of the Pbs2 peptide (IVNKPLPPLPVAGSS) target for Sho1 [9] and the Cla4 peptide target (AHFQPQRTAPKPPIS) for Nbp2 [50] both intensity and binding fraction rank the peptides in top positions. Residues in the SI alignment that have a dark border are highlighted as being conserved and unique within the family.

We calculated the average SC value for groups of residues that define surface I, II and the rest of the domain to address our hypothesis and probe peptide binding further. Fig 4 provides a summary of average SC values, in addition to the number of conserved residues outside the canonical 60 amino acid domain and analysis from published binding data from 4 diverse fungal species [41]. Domains are listed in descending order of the average SC value in SII. Stinkingly, 24/28 domains have average SII SC values between 1.7 and 3.1 and the remaining 4 domains still have values above 1 (between 1.4 and 1.7). In comparison, the average SI SC values are lower (1.0-1.7) which indicates for the overwhelming majority, SII appears more suitable than SI to provide a unique interaction with its peptide target. Interestingly, the domains with higher average SII SC values also had more conserved insertions in the RT-loop and N-Src loop, which may contribute to additional binding specificity. Unique conservation was also observed outside of SI and SII, 9 domains have an average SC value for the remaining residues above 1.7. Furthermore, many domains have uniquely conserved residues in the termini which have been shown to contribute to additional structure to the SH3 domain fold [31, 49]. Overall, the average SC value for these different regions shed more light onto the function of the domains and reveals the majority have the capacity for intrinsic binding specificity.

### Specificity predictions are supported by binding data

To test the accuracy of our binding specificity predictions from the alignments, we calculated a domain-species binding fraction (Eq (2)) for each peptide in a given species. This was calculated from published binding data between 300 peptides and the SH3 domain family members from 4 diverse fungal species [41]. For each domain, we counted the number of peptides that had at least one specific domain-peptide interaction (binding fraction > 0.5) and indicated this number in Fig 4 (#_peptides) and S3 File. Also, indicated in Fig 4 is the average (across species) binding fraction for the most specific peptide (BF_specific), the number of species where this peptide is specific compared to the number of species where data was available (species). From this data, most domains were found to bind to at least one peptide specifically that was conserved across species, the exceptions lie at the bottom of the table with domains that have average SII SC values below 1.8 (Bbc1, Cyk2, Hof1, Lsb1 and 2, Bzz1b, Bem1a and Cyk3). This analysis allows for the prediction of the most specific binding peptides for the majority of the domains. Interestingly, the number of specific peptides revealed from the binding data to each domain varied significantly. Among the domains with high average SC values in SII, Boi1, Sho1 and Rvs167 only had 5 or 6 specific peptides, while Myo3/5 and Lsb3/4 had 44 specific peptides identified (with high proline content). It is noted that the 300 peptides used in the SPOT binding assays are not fully representative of the yeast proteome or known to all be biological targets. However, a domain such as Myo3/5 or Lsb3/4 that can bind a peptide target specifically (compared to the other family members), may still be presented a number of similar specific peptide targets, resulting in potential cross reactivity inside the cell. The biological relevance of these findings requires further study, however, our predictions based on average SII SC values are consistent with the experimental binding data.

Fig 4 and S1 File also shows the identity of the residues for SI and SII in *S.cerevisiae*. As expected the SI residues are highly conserved within each ortholog alignment (red color) and very similar between paralogous domains. W36 and P51 are essential to the SH3 domain as noted previously as the top 2 conserved SH3 domain residues [46], and positions 8, 10, 37 and 54 all generally show aromatic residues (tyrosine is most common). For some domains, SI residues are conserved in the ortholog alignment but their identity is different to that of the rest of the family (see residues with dark border in Fig 4). In other cases, SI residues are not conserved even though they are conserved for most family members. These deviations in SI suggest a possible mechanism to change the properties of this canonical binding surface. For example, Bud14 and Fus1 show significant deviations from the normal SI residue identities. In the case of Fus1, it has been shown experimentally that when the Fus1 SI sequence is mutated back to the canonical sequence, an increase in PxxP peptide binding affinity is observed [13]. As such, we consider this deviation from the canonical SH3 domain family sequence as another type of specific conservation that may not be captured using SC values alone.

### SI and SII sequence profiles reveal varying degrees of non-canonical recognition

**Fig 5:**
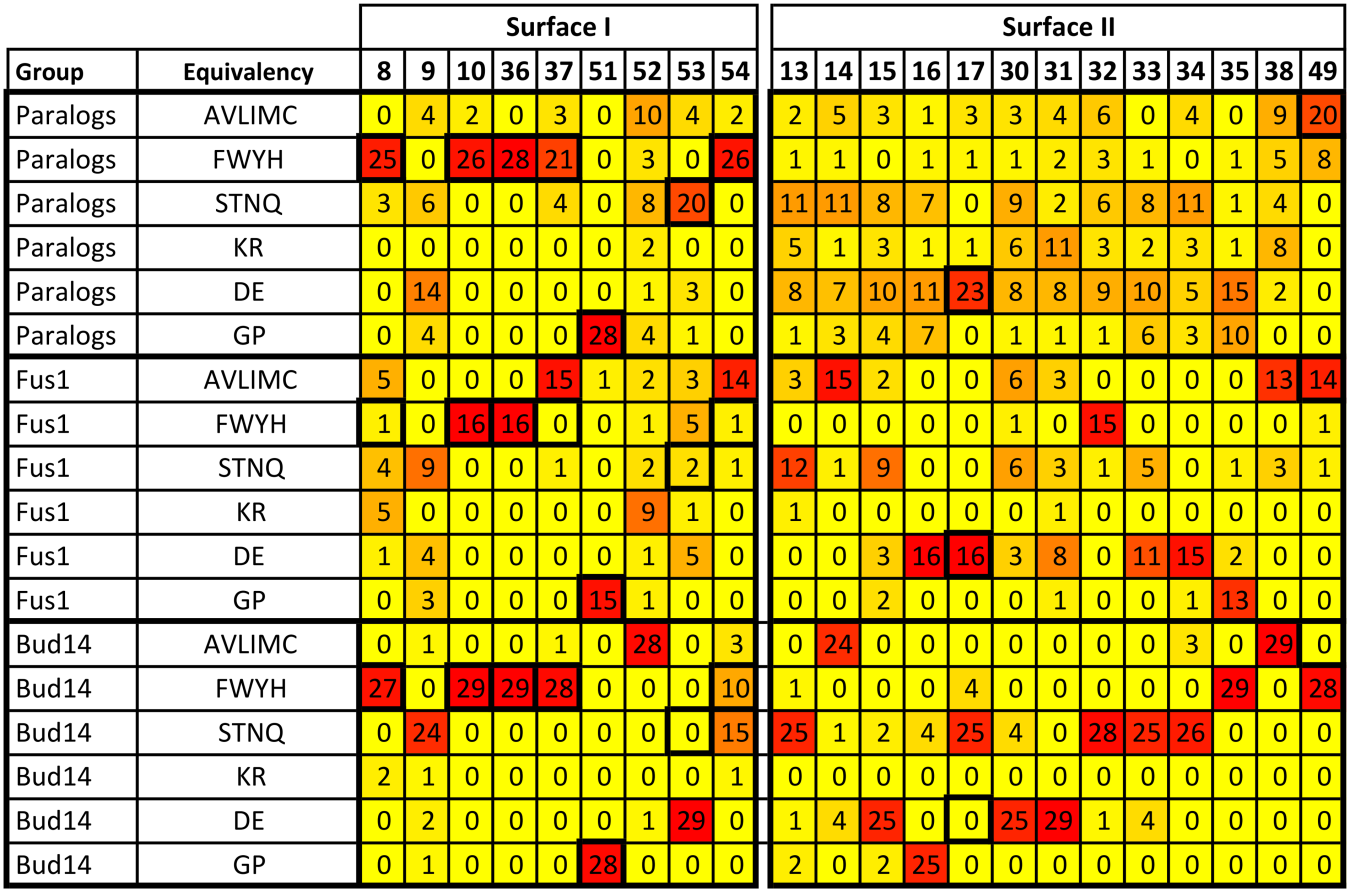
SI and SII PSSM for yeast paralog alignment (28 domains) and example ortholog alignments for Fus1 (16 species) and Bud14 (29 species). Total occurrence for each amino acid group for each position is indicated and colored as yellow (low) to red (high). Residues are grouped into SI (left) and SII (right). Dark outlined regions indicate most common preference for the family (≥ 20 occurrences). Overall, for SI there is a family preference for aromatic residues except the less conserved positions 9, 52 and 53. Notable exceptions include Fus1 that has cysteines at positions 37 and 54 (which are usually in the FWYH group). For SII, there is a loose family preference for polar/acidic residues except at position 49 where hydrophobic residues are found. The extent of conservation in the orthlog alignments in SI and SII vary, with a much greater variation seen in SII PSSMs. PSSMs for all domains (showing both complete domain sequence and only surface I/II) can be found in S4 File.

To complement SC value analysis, we also represent the surfaces in our paralog and ortholog alignments using position specific scoring matrices (PSSMs) to provide additional insights into the family (S4 File). As can be seen in Fig 5, Fus1 and Bud14’s SI deviate from the family sequence and several of these deviations are conserved in its ortholog alignment (indicated by high numbers in deviant positions).

**Fig 6:**
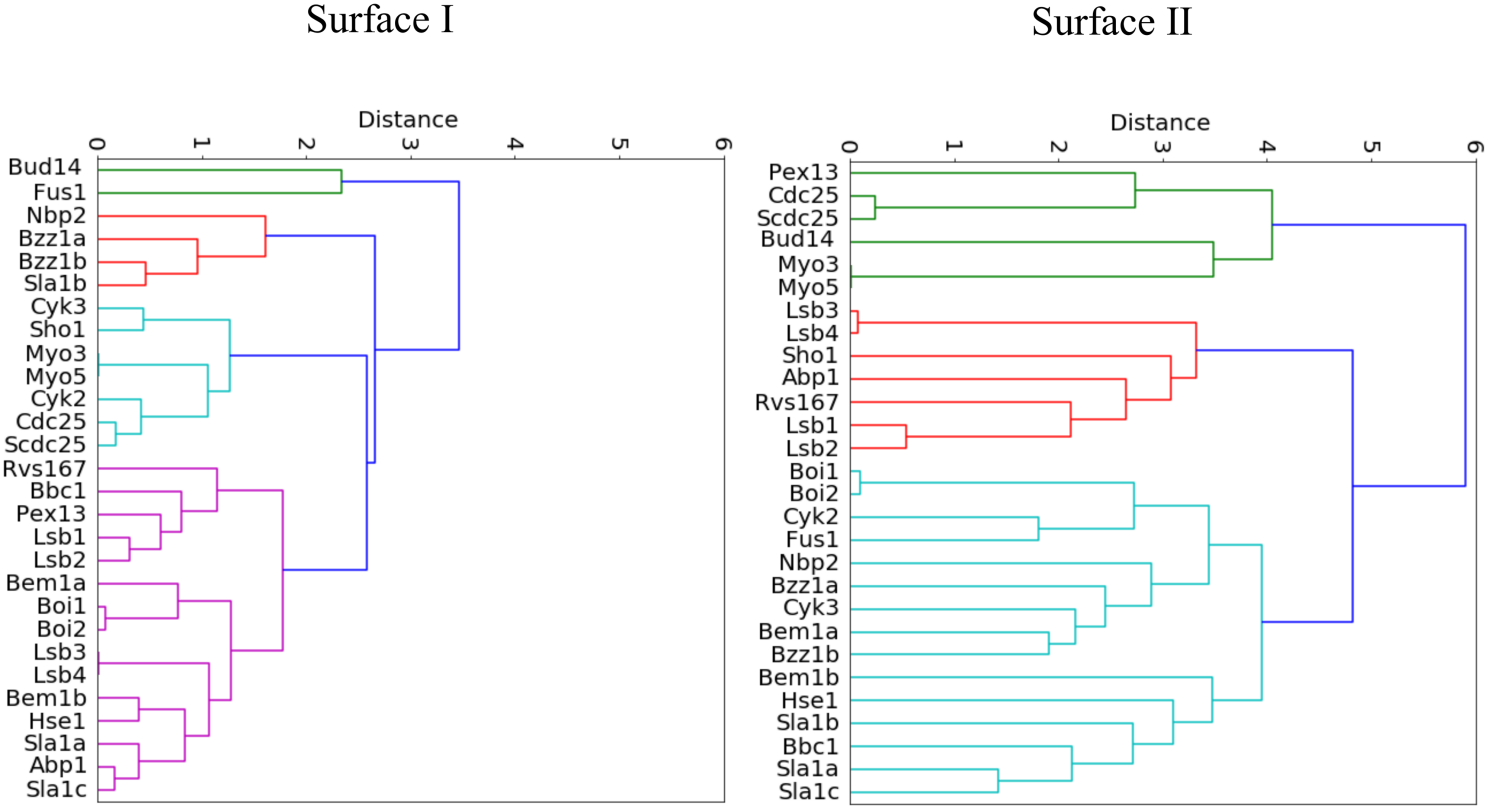
SI and SII family dendograms. Clustering was based on SI (left) and SII (right) PSSMs. For SI dendogram, there is more significant clustering, which appears to concentrate domains that bind class I peptides into the red group and domains that bind class II peptides into the magenta group.

To systematically explore the complete family, we cluster all PSSMs using residues that define SI and find 4 main branches in the resultant dendogram (Fig 6). As expected, SI in Bud14, Fus1 is most distinct (green). For the largest and most conserved group in magenta, 10 out of the 14 domains bind class II peptides (PxxPx+), whereas the less conserved red group contains domains that all bind class I peptides (+xxPxxP) based on phage display studies [40]. The cyan group contains domains that bind both class I and III peptides. Although the differences to the rest of the family are larger for Bud14 and Fus1, the differences in sequence identity and conservation between the class I group (red) and class II group (magenta) are subtle and only differ in 2 or 3 positions. Most interesting is the change in identity for position 37, which is aromatic for class II and aliphatic for class I core motifs. This likely affects the conformation of the adjacent W36, which is a key binding residue for SI (green in Fig 1B).

PSSMs also allow the close examination of diversity in SII, whose residues are chiefly found in loops. As noted previously, SII provides an excellent platform for encoding unique specificity information for a given domain. Figs 4 and 5 show across the family, there is a strong preference in SII for charged/polar residues. This is with the exception of residue position 49, where hydrophobic residues are preferred. A previous study suggested that isoleucine, arginine, valine or methionine at position 49 restricts the conformation of W36 to bind to class II core motifs only, potentially making the domain more intrinsically specific [51]. However, we find these residues in several domains with low average SC SII values (Bem1a, Lsb1, Lsb2, Bbc1) and it appears that predictions of specificity require more than the analysis of a single residue position. As Figs 3 and 5 indicate, yeast domains show unique conservation patterns for most of the family. To comprehensively compare SII sequence profiles for all family members, we also clustered each PSSM by SII to define specificity groups that predict which domains may overlap in peptide binding behavior (Fig 6). The SII dendogram shows that the distances between domains is greater compared to the SI dendogram, indicating SII encodes more variability. Furthermore, there is no correlation between groups in the SII dendogram and peptide class binding preference. Overall, the SII dendogram reveals multiple different specificity groups and suggests almost all domains likely have distinct SII binding surfaces, similar to conclusions reached by phage display studies on human SH3 domains [42].

### 3D analysis of specific conservation reveals additional residue clusters

To complement this analysis, we examined specifically conserved residues for clustering on their 3D structures as well as consider conservation of positions N- and C-terminal of the canonical 60 residue domain boundaries. For 13 of the 28 domains, structures have been deposited in the protein structure database and the remaining 15 core domain structures are easily modeled. Currently, most of these structures await analysis as they were deposited as part of a structural genomics effort. Unfortunately, apart from the Abp1, Nbp2 and Bem1 SH3 domain-peptide complex structures [18, 49, 50], most structures contain either no ligand or short ligands, thus offer limited insight into specific binding requirements. Interestingly, using these structures and our sequence alignment analysis, we identified three other classes of clusters in the domains (besides SI and SII), where several residues with high SC values are near each other on their structure (S2 Fig). First, the core-termini cluster involves specifically conserved residues that make a network from the peptide binding surface through the core to the region where the N- and C-termini meet at the opposite side of the domain (termini region). Second, the termini-distal loop cluster involves surface residues that may form an alternative binding surface. The residues that make this cluster are at the termini region and in the distal loop with additional residues that link these two areas. Finally, some domains have a cluster exclusively at the termini region. In most cases, where the termini region is specifically conserved, the domain also has conserved N and C-terminal extensions (see S2 File) which likely work together. The range of specifically conserved clusters and different degrees of uniqueness in SI and SII in the yeast SH3 domain family suggests that evolution has generated tremendous functional and structural diversity from a common scaffold and a complete analysis of the family will be presented in a future study.

## Discussion

Our simple approach of grouping representative domain sequences into paralog and orthologs allowed us to comprehensively assess the degree of uniqueness for all members of the yeast SH3 domain family. We focused on the binding surfaces I and II, where most proline-rich target peptides bind and large amounts of binding data is available. We also made an initial exploration of other clusters within the domain and sequences outside the canonical boundaries. Our findings, which are supported by high-throughput binding data analysis, accurately predict that most of this family can bind peptide targets specifically via a unique SII [41, 43]. In fact, our study supports two approaches for high intrinsic specificity at the peptide binding site. The first and more common type involves extending the canonical binding region (SI) with a unique surface (SII). A second less common mode changes SI to be unique in addition to a unique SII, making the complete binding surface even more distinct from the other family members (only Bud14, Fus1 and Nbp2 domains significantly deviate from the canonical SI). It should be noted that evolutionary changes occur on a sliding scale as some domains deviate by just one residue in the canonical SI. Thus these two approaches appear to represent two extremes and the family appears to have varying degrees of non-canonical recognition.

Although an extended peptide interaction appears the most common specificity mechanism, we found additional SC clusters and conserved insertions at the loops and termini. This suggests a range of other structural and functional features contribute to the uniqueness of every domain. For example, alternative binding surfaces found in the region from the termini to the distal loop, far from SI and II, could provide contextual specificity mechanisms, especially for domains such as Bem1a and Cyk3. These domains have low average SC values in SII, although an additional interaction at this alternative surface, could facilitate multiple simultaneous target interactions and increased specificity. Furthermore, some domains may increase specificity through their conserved core-termini cluster, which involves a network from the peptide binding site through the core to the termini. For these domains, peptide binding at SI/SII may affect interactions at the termini via allostery, which could be important for specificity [52]. We also consider clusters that may be involved in domain-domain interactions within the same protein, which, for another signaling protein with 2 adjacent domains, confers a binding advantage [53]. For example, in the case of Bem1, based on the presence of several conserved clusters (S2 Fig) in both Bem1a and Bem1b, we predict these domains interact, which will impact target peptide binding. Further study is required to investigate conservation outside of SI/SII to understand their contribution to each domain’s structure and function. However, the yeast SH3 domain family, likely uses almost every mechanism in Fig 1 to attain specificity and fufill its functional role inside the cell. The diversity of specificity mechanisms predicted for the yeast SH3 domain family is similar to the 8 mechanisms described by Das *et al* for functional diversity found over evolution for families of related proteins [54].

Specific conservation and PSSM analysis has been central in addressing our hypothesis that concerns a known binding surface region. The approach has shown functional predictions can be made about protein-protein interaction modules in the absence of peptide binding data and has identified other regions of the domain that could have functional significance. SC values are comparable to positional entropy differences from a previous approach that performed saturation mutagenesis of the binding site between human growth hormone and its receptor [55]. The authors identified/separated specificity determining positions from stability determining positions like our study. Furthermore, several other studies have attempted to take large (super)families of related sequences to identify sub-families and ortholog groups to locate specificity determining sites on proteins [56–62]. Often these approaches are aimed at assigning function to an unknown protein. Our approach simplifies this aim considerably, as we start with a family of known paralogs (from a well annotated model species), with a known phylogenetic tree and general function and construct multiple sequence alignments for each paralog for specific conservation and PSSM analysis. As found in other studies, protein families are nuanced, while the house-keeping residues important for folding or stability or basic functionality are conserved across all ortholog alignments (albeit low SC values), specificity determining residues (high SC values) are often under positive selection for distinct functional properties that go beyond a canonical binding site [60, 63–65].

Difference evolutionary trace (ET) is an alternative to our approach that can also identify important unique specific residues for paralogs [66–68]. Difference ET studies have examined 7 main evolutionary branches (equivalent to paralogs) in the intracellular zinc binding domain family [66] and considered 3 main branches (opsin, serotonin and dopamine receptors) in the G protein-coupled receptor family [67, 68]. Our approach differs from ET as it forms ortholog alignments using similar or identical species for every family member, ensuring a fair, simple comparison of members within a family (and needs little normalization). As such, it is easily scalable to larger domain families such as the human SH3 domain family (>300 members) or other domain families in the fungal kingdom or other kingdoms of interest. We anticipate many more structural and functional insights for domain families using this simple approach. From this study, it is clear that SH3 domains are far from passive proline rich docking domains and instead have conferred active functional evolution towards almost every one of its 60 residues.

## Supporting information

S1 Table Taxonomy groups for ortholog alignments. *Sacharamyces cerevisiae* has a group number of 0, all other saccharamycotina species have a group number of 1. Group numbers are indicated in parentheses (see S1 Fig) and are found in alignment files S2 and S1 Files. The number of species that ideally represent each group in the ortholog alignments is indicated in the last column.
(TIF)

S2 Table Species paralog count table. Number of species available at each taxonomic level (S1 Table) for direct relatives (orthologs) of each SH3 domain family member in our alignments.
(TIF)

S1 Fig Yeast phylogenetic tree highlighting taxonomic groups. A tree depicting the relationships between the fungal species groups (S1 Table) compared in our ortholog alignments. The branching pattern indicates which species are most closely related to each other. The length of the branches is not proportional to phylogenetic distance or to divergence time. This tree was constructed according to a variety of published phylogenies [69–71]. Indicated on the right hand side is the number of species we select from each group to maximize diversity in our alignments.
(TIF)

S2 Fig Yeast domain family sequence conservation summary. Domains are sorted in descending order by their average SII SC value. Table shows number of additional conserved residues either N- and C- terminal to the SH3 domain or insertions in the 3 loops (highlighted in red) as well as significant SC found in the binding surface and other residue clusters (indicated by an X). The core-termini cluster usually involve residues 37,50 and 52 as well as residues in the termini, thus potentially connecting binding to changes near the termini via coupled conformational changes. The termini-distal cluster is a group of surface residues, typically involving residues 25 and 27 that connect the distal loop to the termini residues. The termini cluster includes the termini residues as well as other surface residues to form another binding surface. For some domains alternative names are provided in parentheses.
(TIF)

S1 File Sequence conservation analysis for orthologous SH3 domains. **(Stacked Domains Tab)**. Only SH3 domain residues that align with the *S.cerevisiae* domain are shown and are colored according to our residue equivalence groups defined for entropy and PSSM calculations. The species names end with a number that refers to their taxonomic group as seen in our tree (S1 Table). The SC value is calculated as paralog entropy/ortholog entropy. A standard numbering system [46] for the core 60 SH3 domain residues is indicated on the top row as well as the residue number in the full length *S.cerevisiae* protein (fifth row). The paralog entropy is calculated from an alignment of the 28 SH3 domains in *S.cerevisiae* and is found at the bottom. (Paralog Align Tab). Full alignment of the yeast SH3 domain family. (Individual SH3 domain Tabs). Each yeast SH3 domain is aligned with its Fungal orthologs. Only SH3 domain residues that align with the *S.cerevisiae* domain are shown.
(XLSX)

S2 File Sequence conservation analysis of orthologous full length proteins. Full-length SH3 domain containing proteins are aligned. Each tab contains the alignment of one yeast SH3 domain containing protein and all of its direct relatives. The 1st row contains the alignment numbers, the 2nd row, the ortholog entropy, which is only calculated if the no gap proportion (3rd row) is above its threshold (0.64). The 4th row contains the standard domain number. The 5th row contains the residue number according to the yeast protein. Residues are colored according to our residue equivalence groups defined for entropy and PSSM calculations. The species names end with a number that refers to their taxonomic group as seen in our tree (S1 Table).
(XLSX)

S3 File Specific binding peptides for the yeast SH3 domain family. Peptide sequences for a peptide that has a binding fraction > 0.5 in at least one species. Binding fractions <0.5 are shaded green. The top peptide for each domain has its average binding fraction highlighted yellow.
(XLSX)

S4 File PSSMs for the yeast SH3 domain family. Total occurrence for each amino acid group for each position is indicated and colored as yellow (lowest value) to red (highest value).
(XLSX)
