# Supplementary figures and images for: Most yeast SH3 domains bind peptide targets with high intrinsic specificity
Source: PLoS One. 2018 Feb 22;13(2):e0193128. doi: 10.1371/journal.pone.0193128 (PMC5823434; doi:10.1371/journal.pone.0193128)

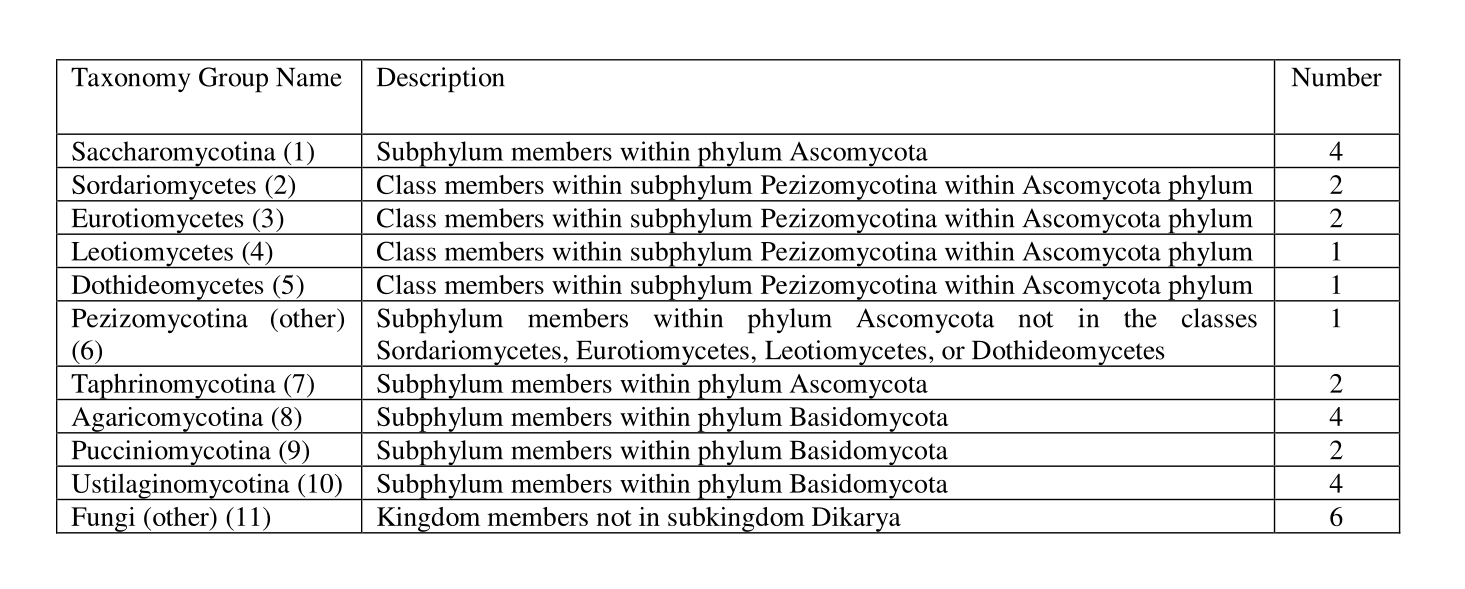

Supplement: S1 Table — Sacharamyces cerevisiae has a group number of 0, all other saccharamycotina species have a group number of 1. Group numbers are indicated in parentheses (see S1 Fig) and are found in alignment files S2 and S1 Files. The number of species that ideally represent each group in the ortholog alignments is indicated in the last column. (TIF) [file pone.0193128.s001.tif]

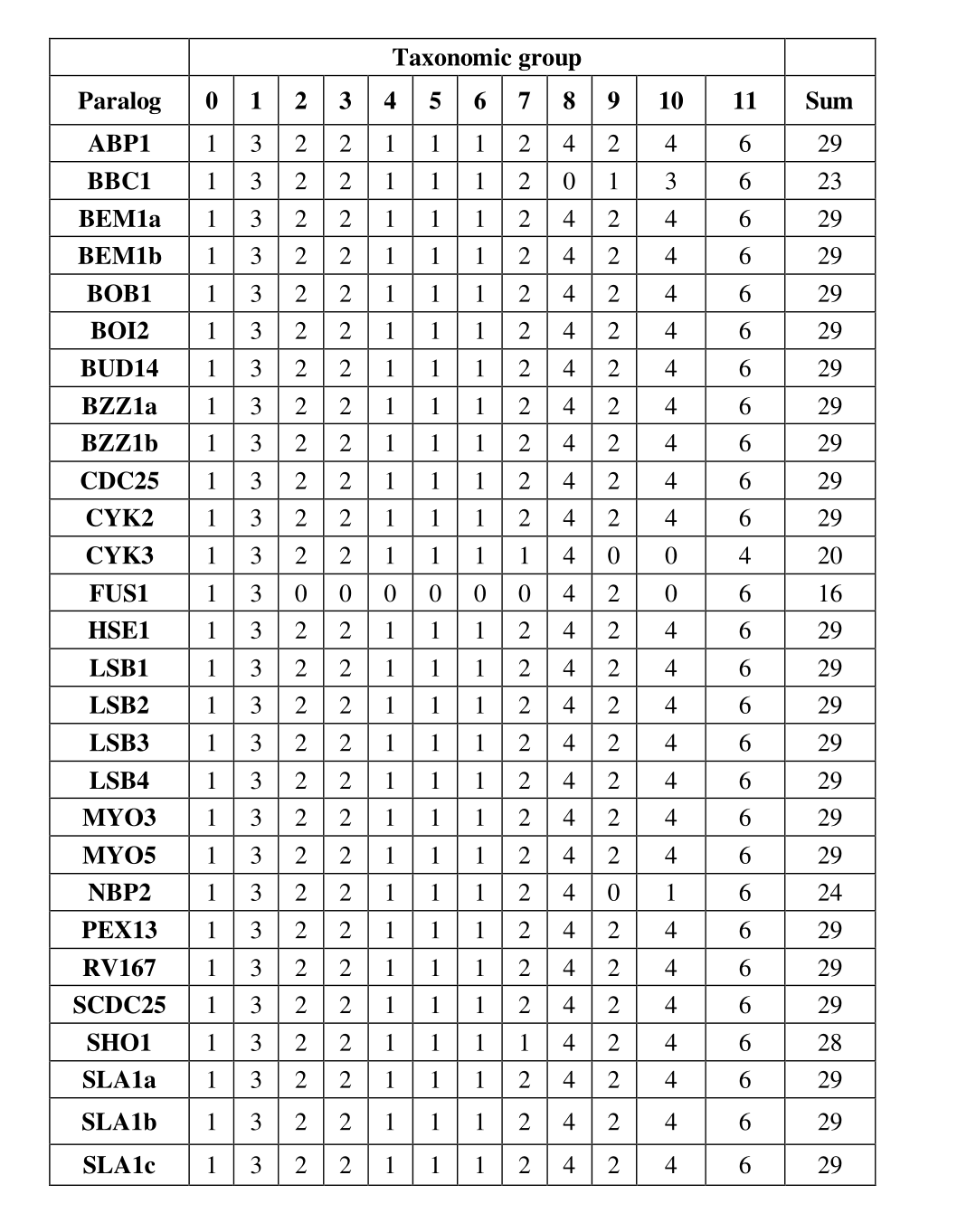

Supplement: S2 Table — Number of species available at each taxonomic level (S1 Table) for direct relatives (orthologs) of each SH3 domain family member in our alignments. (TIF) [file pone.0193128.s002.tif]

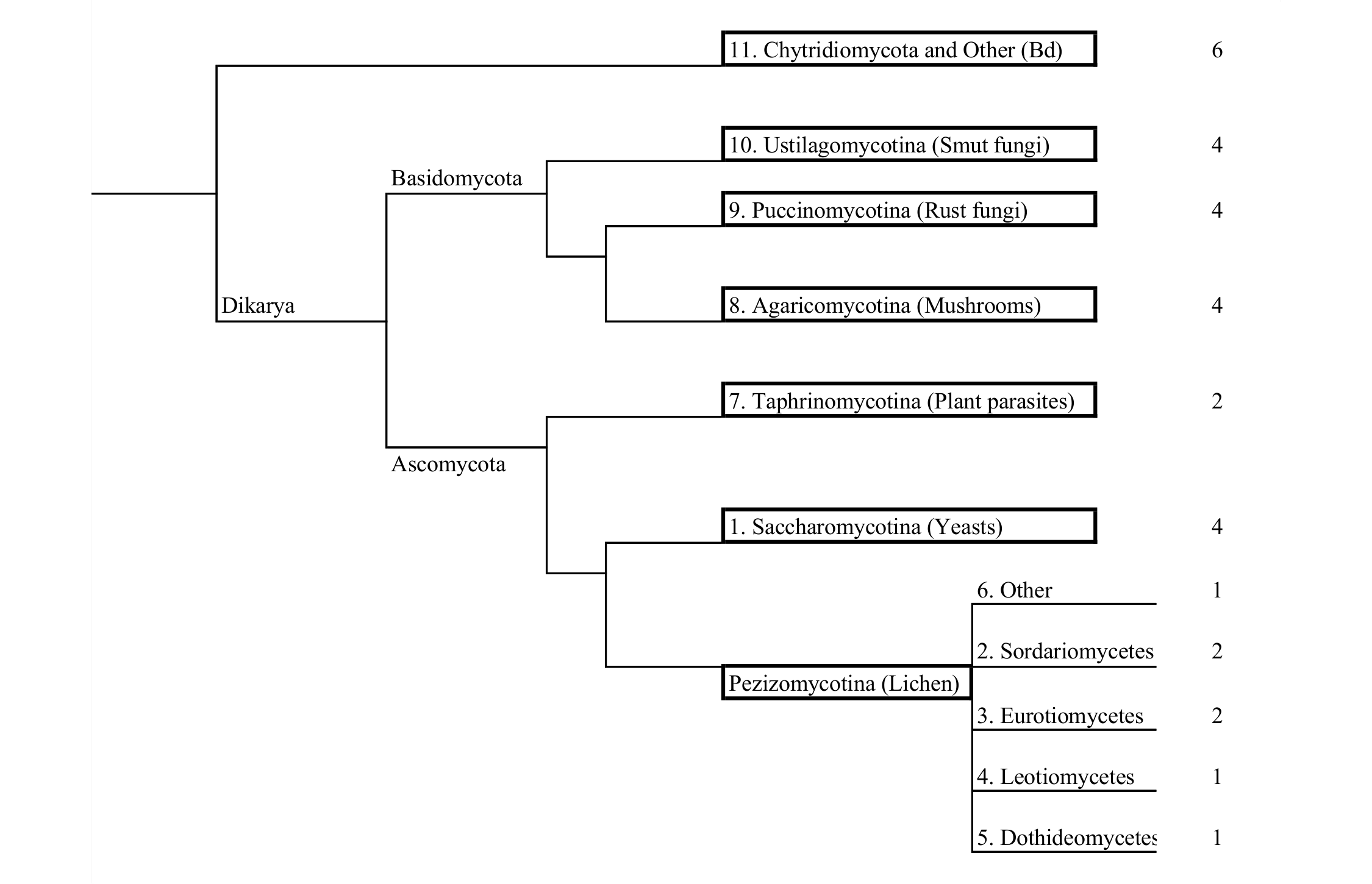

Supplement: S1 Fig — A tree depicting the relationships between the fungal species groups (S1 Table) compared in our ortholog alignments. The branching pattern indicates which species are most closely related to each other. The length of the branches is not proportional to phylogenetic distance or to divergence time. This tree was constructed according to a variety of published phylogenies [69–71]. Indicated on the right hand side is the number of species we select from each group to maximize diversity in our alignments. (TIF) [file pone.0193128.s003.tif]

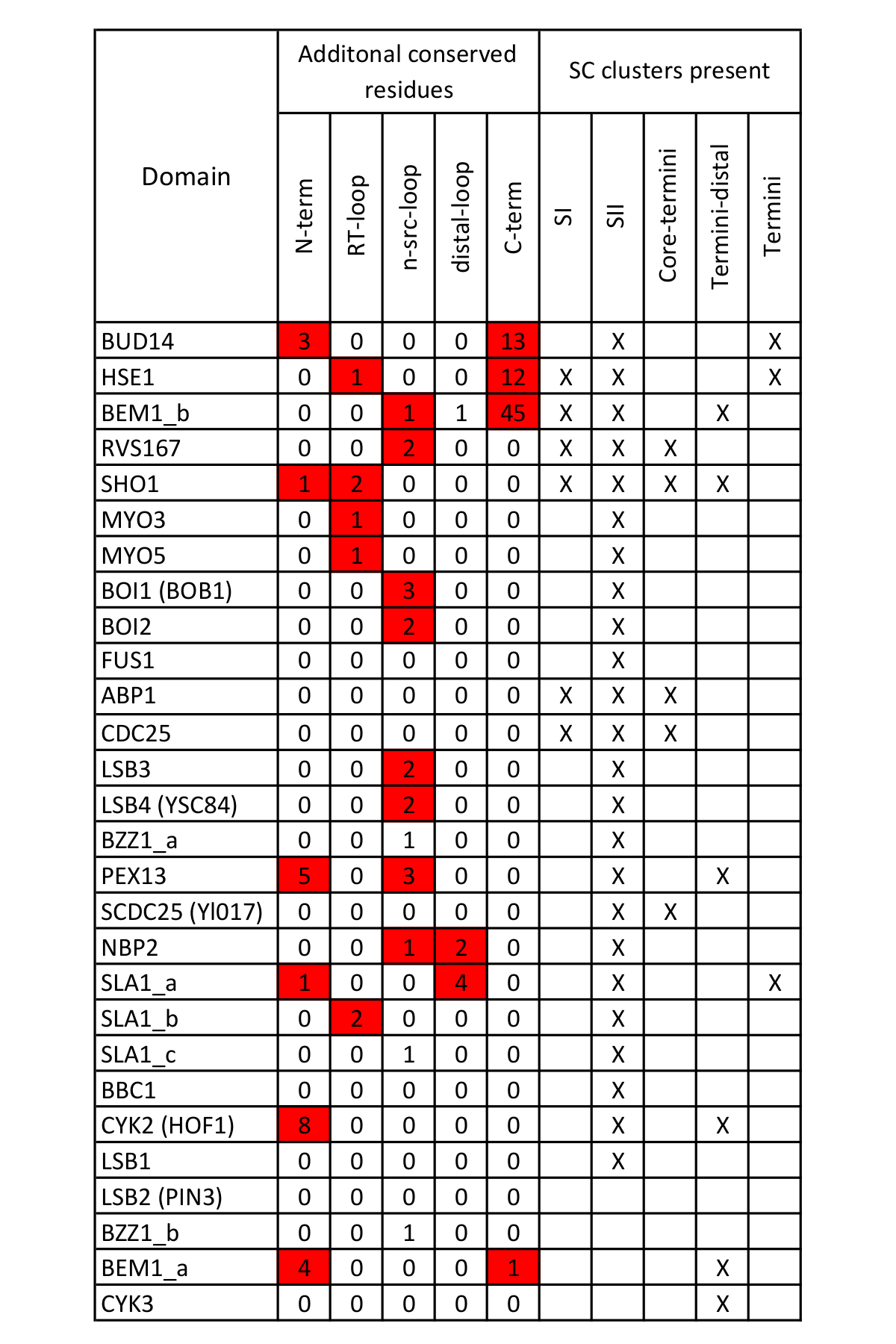

Supplement: S2 Fig — Domains are sorted in descending order by their average SII SC value. Table shows number of additional conserved residues either N- and C- terminal to the SH3 domain or insertions in the 3 loops (highlighted in red) as well as significant SC found in the binding surface and other residue clusters (indicated by an X). The core-termini cluster usually involve residues 37,50 and 52 as well as residues in the termini, thus potentially connecting binding to changes near the termini via coupled conformational changes. The termini-distal cluster is a group of surface residues, typically involving residues 25 and 27 that connect the distal loop to the termini residues. The termini cluster includes the termini residues as well as other surface residues to form another binding surface. For some domains alternative names are provided in parentheses. (TIF) [file pone.0193128.s004.tif]
